# Phase-driven progress in nanophotonic biosensing

**DOI:** 10.1038/s41377-024-01415-3

**Published:** 2024-03-18

**Authors:** Isabel Barth, Hakho Lee

**Affiliations:** Center for Systems Biology, Massachusetts General Hospital, Harvard Medical School, Boston, MA 02114 USA

**Keywords:** Optical sensors, Imaging and sensing, Nanophotonics and plasmonics

## Abstract

In the continuous pursuit of enhancing the sensitivity of nanophotonic biosensors by leveraging phase phenomena, a recent development involved the engineering of an atomically thin Ge_2_Sb_2_Te_5_ layer on a silver nanofilm to generate large Goos–Hänchen-shifts associated with phase singularities. The resulting detection limit reached ~7 × 10^−7^ RIU.

Early developments in nanophotonic biosensing focused on exploiting the unique optical properties of nanomaterials, such as plasmonic nanoparticles and photonic crystals^1^, to enable label-free and real-time monitoring of biological interactions. Leveraging phenomena like surface plasmon resonances and whispering gallery modes to detect minute changes in refractive index now allows the detection of biomolecular interactions at the single-molecule level with implications for clinical diagnostics. Recent technical advances^2^ include the integration of metamaterials and advancements in fabrication techniques, like nanoimprint lithography, which have enabled the development of low-cost, compact and portable biosensing devices.

Simultaneously, there is an ongoing quest to further enhance the sensitivity of nanophotonic biosensors, especially by exploiting phase phenomena associated with photonic resonances. The goal is to enable label-free sensing technology with a maximized refractive index resolution while reducing the requirements in nanofabrication, setup complexity, and cost.

In recent decades, successful applications of detection schemes utilizing spectral or angular information, along with intensity-based read-out approaches as transducers, have been observed in label-free biomolecular detection using nanophotonic sensors. These advancements have led to the development of various platforms that exhibit competitive or, in some cases, superior, sensitivity compared to the diagnostic standard ELISA. However, improving the performance typically entailed a relatively high system complexity. Fortunately, new alternative approaches promise to achieve high sensitivity in simpler platforms by exploiting steep phase responses.

Two parallel paths are being explored for phase-driven progress:

1. Direct phase interrogation: This method uses interferometry to directly interrogate photonic resonances. It has shown promise in both plasmonic^3^ and dielectric^4^ platforms, offering enhanced sensitivity and potential for miniaturization.
2. Phase-enhanced, indirect detection: Another approach leverages phase phenomena, for instance, enhanced intensity-based detection. Recently, there has been significant interest in topologically protected phase singularities^5,6^ and amplified Goos–Hänchen-shifts^7^ for biosensing.

In their recent paper published in Light Science & Applications (LSA), Zhu et al.^8^ have achieved significant progress in Goos–Hänchen (G–H)-shift-based sensing. Their method specifically generated singularized phase responses through the integration of an atomically thin layer of Ge_2_Sb_2_Te_5_ (GST) on a silver nanofilm. G–H-shifts are small lateral beam displacements associated with total internal reflection and interference of phase-shifted components of the beam, leading to a dependence of the shift on the refractive index at the interface due to evanescent waves. Since the G–H shift is proportional to the phase shift differences acquired upon total internal reflection, the small beam displacements that are acquired with single-layer dielectric interfaces can reach hundreds of microns due to steeper phase responses arising from resonances and enhanced absorption with additional materials.

Zhu et al.^8^ show that the phase singularity associated with the engineered absorption in the GST layer resulted in a steep phase curve (Fig. 1b) and unprecedented position shifts of up to ~440 µm, which is higher than previously achieved even with similar approaches. Since the authors target their technology at medical diagnostics beyond typical laboratory settings, they characterized the noise of their system in addition to the sensitivity, demonstrating a limit-of-detection (LOD = Noise/Sensitivity) of ~7 × 10^−7^ RIU. This result is superior to several simpler direct phase interrogation approaches (LOD down to ~10^−6^ RIU) and close to some direct phase-sensitive SPR modalities (resolution ~10^−8^ RIU). However, it should be noted that some of these reported findings in phase-sensitive modalities are not based on the above-defined LOD with its proportionality to the system noise, which, in an un-stabilized biosensing system with temperature- and mechanical drift, can greatly surpass the theoretical phase-resolution.

**Fig. 1:**
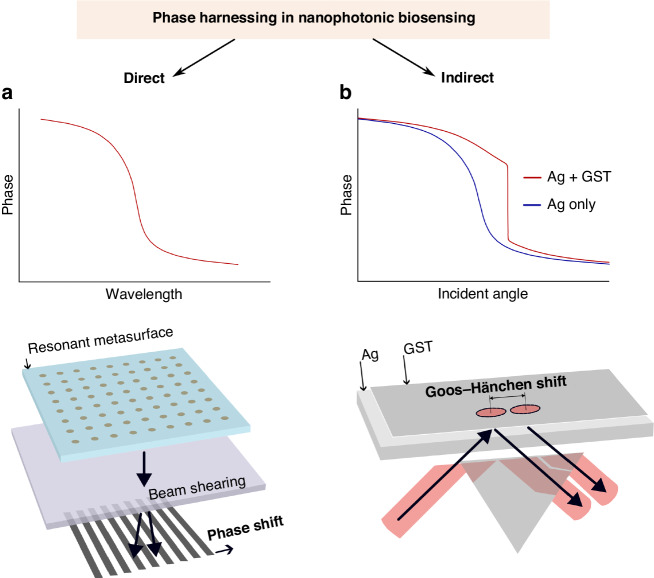
Singular-phase-enhanced Goos–Hänchen shift in the context of the indirect and direct harnessing of phase behavior in nanophotonic biosensing. **a** Schematic of phase behavior and direct phase interrogation with polarization beam shearing, based loosely on a phase-sensitive plasmonic biosensor^3^ and an interferometric dielectric platform^4^. **b** Schematic based on Zhu et al.^8^

Zhu et al.^8^ further verified the label-free detection of small cytokine biomarkers (e.g., TNF-α, 17.3 kDa) down to a concentration of 0.1 fM, which represents an order of magnitude improvement compared to similar approaches. For future validation, however, these proof-of-principle detection limits would need to be confirmed in complex matrices, such as serum, with adequate biochemical controls.

A relevant difference between sensors based on, for instance, plasmonic and dielectric nanohole arrays^9^ and on G–H shift detection is the imaging capability. While hyperspectral imaging, intensity-based or direct interferometric approaches^3,4^ with resonant metasurfaces, allows imaging of refractive index distributions on the sensor surface and imaging-based, multichannel detection^10,11^, G–H-shift-based platforms are currently not imaging-based. Despite the impressive sensitivity, the simultaneous detection of multiple biomarkers for sufficient clinical accuracy with high throughput represents one of the challenges going forward.

## References

[1] Pitruzzello, G. & Krauss, T. F. Photonic crystal resonances for sensing and imaging. *J Opt*. **20**, 073004 (2018).

[2] Altug, H. et al. Advances and applications of nanophotonic biosensors. *Nat. Nanotechnol.* **17**, 5–16 (2022). 10.1038/s41565-021-01045-5 35046571

[3] Yesilkoy, F. et al. Phase-sensitive plasmonic biosensor using a portable and large field-of-view interferometric microarray imager. *Light Sci. Appl.* **7**, 17152 (2018). 10.1038/lsa.2017.152 PMC6060062 30839537

[4] Barth, I. et al. Common-path interferometric label-free protein sensing with resonant dielectric nanostructures. *Light Sci. Appl.* **9**, 96 (2020). 10.1038/s41377-020-0336-6 PMC7264974 32509300

[5] Ermolaev, G. et al. Topological phase singularities in atomically thin high-refractive-index materials. *Nat. Commun.* **13**, 2049 (2022). 10.1038/s41467-022-29716-4 PMC9019097 35440544

[6] Kabashin, A. V., Kravets, V. G. & Grigorenko, A. N. Label-free optical biosensing: going beyond the limits. *Chem. Soc. Rev.* **52**, 6554–6585 (2023). 10.1039/d3cs00155e 37681251

[7] Wang, Y. Y. et al. Targeted sub-attomole cancer biomarker detection based on phase singularity 2D nanomaterial-enhanced plasmonic biosensor. *Nano-Micro Lett.* **13**, 96 (2021). 10.1007/s40820-021-00613-7 PMC7985234 34138312

[8] Zhu, S. D. et al. Label-free biosensing with singular-phase-enhanced lateral position shift based on atomically thin plasmonic nanomaterials. *Light Sci. Appl*. **13**, 2 (2024). 10.1038/s41377-023-01345-6 PMC10757996 38161210

[9] Conteduca, D. et al. Dielectric nanohole array metasurface for high-resolution near-field sensing and imaging. *Nat. Commun.* **12**, 3293 (2021). 10.1038/s41467-021-23357-9 PMC8172834 34078903

[10] Kenaan, A. et al. Guided mode resonance sensor for the parallel detection of multiple protein biomarkers in human urine with high sensitivity. *Biosens. Bioelectron.* **153**, 112047 (2020). 10.1016/j.bios.2020.112047 31999559

[11] Tittl, A. et al. Imaging-based molecular barcoding with pixelated dielectric metasurfaces. *Science* **360**, 1105–1109 (2018). 10.1126/science.aas9768 29880685

